# Biology and survival of extremely halophilic archaeon *Haloarcula marismortui* RR12 isolated from Mumbai salterns, India in response to salinity stress

**DOI:** 10.1038/srep25642

**Published:** 2016-05-27

**Authors:** Rebecca S. Thombre, Vinaya D. Shinde, Radhika S. Oke, Sunil Kumar Dhar, Yogesh S. Shouche

**Affiliations:** 1 Department of Biotechnology, Modern College of Arts, Science and Commerce, Shivajinagar, Pune-411005. Maharashtra, India; 2 Microbial Culture Collection (MCC), National Centre for Cell Science, Ganeshkhind, Pune, 411007, India

## Abstract

Haloarchaea are unique microorganism’s resistant to environmental and osmotic stresses and thrive in their habitats despite extreme fluctuating salinities. In the present study, haloarchaea were isolated from hypersaline thalossohaline salterns of Bhandup, Mumbai, India and were identified as *Haloferax prahovense*, *Haloferax alexandrines, Haloferax lucentense, Haloarcula tradensis, Haloarcula marismortui* and *Haloarcula argentinensis*. The mechanism of adaptation to contrasting salinities (1.5 M and 4.5 M) was investigated in the extreme haloarchaeon, *Hal. marismortui* RR12. *Hal. marismortui* RR12 increased the intracellular sequestration of K^+^ and Cl^−^ ions in hypo salinity and hyper salinity respectively as detected by Energy-dispersive X-ray spectroscopy microanalysis (EDAX) and Inductively Coupled Plasma- atomic Emission Spectroscopy (ICP-AES) indicating the presence of ‘salt-in’ strategy of osmoadaptation. As a cellular response to salinity stress, it produced small heat shock like proteins (sHSP) identified using MALDI-TOF MS and increased the production of protective red carotenoid pigment. This is the first report on the study of the concomitant cellular, molecular and physiological mechanism adapted by *Hal. marismortui* RR12 when exposed to contrasting salinities in external environment.

The ever-increasing anthropogenic activities as well as extreme changes in the environment resulting due to global warming have started affecting the survival of microbial life in their ecological niches and habitats. Most micro organisms are not able to survive rapid changes in the environment as they require optimum conditions for growth. However, extremophilic organisms are able to adapt, adjust and survive in extreme flux in environmental growth conditions and parameters. ‘Halophiles’ are such salt loving extremophilic organisms requiring 1.5 to 5 M salt concentration for their growth and survival^1^. Haloarchaea are halophilic organisms belonging to the domain archaeabacteria, which have a strict requirement of minimum 1.5 mol l^−1^ sodium chloride for growth^2^. The first description of the red halophilic prokaryote was reported in 1919 by Henrich Kleban^3^. The *Halobacteriaceae* family till date comprises of 36 genera and 129 species^4^. These organisms are known to dwell in hypersaline marshes, solar salterns-manmade and naturally occurring, saline ponds and some have also been isolated from low salinity environments. Haloarchaea are not only exposed to salinity changes, but are also predisposed to very strict conditions of growth in presence of UV radiations, high ionic stresses, high temperature and alkaline pH. They have hence adapted and evolved various mechanisms to survive the environmental perturbations by the means of ‘osmoadaptation’ strategies. In addition to osmoadaptation, haloarchaea adapt to adverse environmental conditions by developing specific cellular and molecular responses. In particular, the universally known heat shock response occurs when cells are exposed to elevated temperatures, resulting in the rapid and transient overproduction of a limited class of proteins called the heat shock proteins (HSP’s), small heat shock proteins (sHSP’s) and other stress factors. The HSP’s produced in the domains archaea are highly conserved both in structure and function, and their induction is generally regulated at the transcription initiation level^5^. However, the physiological effects of ionic stress remain largely unexplored. The classical adaptation strategies so far accepted for salinity stress are the “Salt-in strategy” where the haloarchaeon sequesters the cations (sodium or potassium) inside the cytosol so as to maintain the ionic concentration in the cell equivalent or higher than the external environment^6^. The organisms employing the salt -in strategy have a predominance of acidic charged proteome and most organisms adapting this mechanism utilize the Na^+^/H^+^ antiporters and ATPase dependent ion transporters for the stable maintenance of sodium gradient across the cell. Thermodynamically, the organism has to utilize the proton motive force to maintain the ionic gradient. The second strategy is the production of organic or compatible solutes. This strategy is largely known not only in Domain Archaea but also in the Domain Bacteria and Domain Eukarya. Organisms accumulate organic solutes by uptake from environment or de novo synthesize organic compounds like sugars and polyols (glycerol, sucrose and trehalose, sorbitol, mannitol), amino acid derivatives, and compatible solutes (glycine-betaine, ectoine and hydroxyl ectoine) for protection against salinity stress^7^. There are many studies regarding compatible solute strategy in Haloarchaea^8^,^9^,^10^,^11^, however studies related to “salt -in strategy” during contrasting salinity stress in haloarchaea are restricted. Major studies have reported the isolation, characterization of Haloarchaea for exploration of their biotechnological potential, however studies related to investigation of the survival strategies adapted by these organisms in fluctuating salinities in their eco-niches is uncommon. Haloarchaea in India have been previously isolated from natural solar salterns of Ribander, Goa^12^ and coastal and marshy areas of Gujarat^13^,^14^,^15^. Our group has previously reported isolation of extremely halophilic archaea from Kanjurmarg salterns, Mumbai^16^ and isolation of *Hfx. mediterranei* from low salinity environment of the sea water of Arabian sea lining the area of Marine drive, Mumbai, India^1^,^17^. In the present investigation we have isolated haloarchaea from the solar saltern of Bhandup area of Mumbai, India. The Bhandup area is busy suburban area within Greater Mumbai and the Bhandup-Nahur belt has solar salterns which have not been previously explored for the isolation of halophiles. We have isolated 34 haloarchaea and based on morphological and physiological characteristics identified nine haloarchaea from this saltern. To the best of our knowledge, the isolation of archaeal strains of *Hal. tradensis* RR14, *Hal. argentinensis* RR10*, Hfx. lucentense* RR15 and *Hfx. prahovense* RR9 are being reported for the first time from salterns of Mumbai. The concentration of salt fluctuates rapidly in the saltern at various stages of crystallization and evaporation due to seasonal changes like heavy monsoon and summer. Despite these changes, haloarchaea have to adapt and survive in such unstable salinity concentrations. Prior studies have provided insights in genomic or proteomic basis of stress survival in bacteria and halophiles^18^,^19^,^20^,^21^,^22^. However, there is meager data available on the physiological, cellular and osmoadaptation strategies of the haloarchaeon, *Haloarcula marismortui.* This is presumably the first report on the study of physiological and cellular adaptation of *Hal. marismortui* RR12 isolated from solar salterns of Mumbai, India to ionic stress in response to sodium chloride.

## Methods

### Collection of sample and isolation of haloarchaea

Soil and brine samples were collected from solar salterns present in Bhandup area (19.1300° N, 72.9400° E), Mumbai, India in sterile plastic container during February 2014. The sample was analyzed for Na, K, Mg and Cl content using standard methods^23^. The soil sample was enriched in Sehgal and Gibbon’s medium (SG) containing (g/L) Casamino acids- 7.5; Yeast Extract- 10, potassium chloride- 2, trisodium citrate- 3, MgSO_4_- 20; NaCl- 150; pH- 7.2 at 40 °C for 7 days at 100 rev min ^−1^ in a rotary shaking incubator until intense pink coloration was observed^1^. A loopful from the enrichment broth was isolated on SG medium containing 15% NaCl after typical red colonies appeared they were sub cultured on SG slants and stored as glycerol stocks at −20 °C till future use.

### Identification of haloarchaea using morphological, biochemical and physiological test

Identification and characterization studies of the isolated organisms were performed as per the minimal standard for the description of the taxa in the *Halobacteriales* order^24^,^25^. The gram reaction of the organisms was studied as described by Dussault^26^. The optimum concentration of sodium chloride required for growth was tested by inoculating the organisms in SG medium containing different salt concentrations ranging from 0, 0.34, 0.85, 1.19, 1.36, 1.54, 1.71, 2.56, 3.42, 4.28, 5.13 and 5.99 mol l^−1^ NaCl at 40 °C for 7–15 days. Similarly, the optimum pH and temperature of the haloarchaea were assessed as described earlier. Detection of catalase activity was performed by slide catalase test using 1% (v/v) hydrogen peroxide. Visibility of effervescence confirmed the catalase positive test for that organism. No effervescence confirmed negative catalase test. Detection of Cytochrome oxidase activity was assessed by spotting the culture on whatmann no. 1 filter paper and adding few drops of oxidase reagent (Himedia, Mumbai, India) to it. Change of the color of the culture from pink to blue confirmed the presence of cytochrome oxidase. Utilization of sugar, amino acids and production of nitrate reductase, urease, gelatinase, protease, lipase and amylase was assessed as described earlier by our group^16^. The IMViC tests were performed as described by Azhar *et al.*^27^.

### 16S rRNA gene sequencing of Haloarchaea

The haloarchaeal cultures were identified using 16S rRNA gene sequencing method as described by Sharma *et al.*^28^. The genomic DNA was isolated using a DNA isolation kit (Invitrogen) and the 16S rRNA gene sequence was amplified using the ARC20F: TTCCGGTTGATCCYGCCRG and ARC958R: YCCGGCGTTGAMTCCAATT archaeal primers. The DNA sequence covered with these primers was around 800 bps. The PCR product was then purified using a Rapid Tip kit from Diffinity Genomics and DNA sequencing was carried out using an ABI PRISM Big Dye Terminator v3.1 Cycle Sequencing kit on a 3730× l Genetic Analyzer (Applied Biosystems). The sequence data were edited with the Seqman DNA Star software. The taxonomic identity of the 16S rRNA gene sequence of strains and its percentage similarity with other taxa was calculated using the ‘Identify’ option of the EzTaxon-e server^29^. The 16S rRNA gene sequences of all the haloarchaea were deposited in NCBI GenBank with accession numbers KP712893, KP739945, KP712898, KP712899, KP739946, KP712894, KP712895, KP712896, KP712897 and KP739947 for isolates RR8-RR17. The haloarchaeal isolates were also deposited in Microbial Culture Collection (MCC), National Centre for Cell Sciences, Pune, India.

### Effect of sodium chloride concentration on growth haloarchaea

The effect of NaCl concentration on growth of haloarchaea was studied by monitoring its growth in SG medium containing 5%, 8%, 10%, 15%, 20%, 25%, 30% & 35% (w/v) NaCl at 40 °C for 7 days. The growth was measured in terms of absorbance at 600 nm using UV-Visible Spectrophotometer (Shimadzu, Japan). The isolate RR12 (*Hal. marismortui*) that demonstrated growth and stable pigment production over a wide range on sodium chloride concentration (8% to 20% NaCl) was selected for further studies.

### Effect of salinity stress on growth of *Hal. marismortui* RR12

For assessing the effect of salinity stress on *Hal. marismortui* RR12, the organism was cultured in SG broth containing 15% NaCl (2.5 M) concentration at optimum conditions and thereafter centrifuged at 10 000 ×  *g* for 0.5 h. The cell pellet was inoculated in SG medium with low salinity (1.5 M NaCl) and high salinity (4.3 M NaCl)^30^ and incubated at 40 °C for 48 h at 100 rev min^−1^ in an orbital shaker. After the salinity shock treatment, the cells were centrifuged, the supernatant was discarded and the stressed cells were used for further physiological studies.

### Preparation of cell lysate and 1D- SDS PAGE, Trypsin digestion & In- gel extraction of peptides for MALDI

After exposure to salinity stress, the haloarchaea was subjected to centrifugation at 10,000 ×  *g* for 30 min at 4 °C. The supernatant was discarded and the cell pellet was treated in gel loading buffer as described previously^1^. The soluble fraction of extracted proteins was subjected to separation using SDS-PAGE^31^. The differentially expressed protein bands observed in the stressed samples resolved through polyacrylamide gel electrophoresis were identified and selected for trypsin digestion. These bands were excised from the gel and washed repeatedly with water and 50% acetonitrile for 15–20 min. The gel pieces were then heated with equi-volume mixture of 100 mM NH_4_HCO_3_ and acetonitrile for 15 min. After that the gel pieces were soaked in solution containing 10 mM DTT and 0.1 M NH_4_HCO_3_ for 45 min to 50 min at 56 °C followed by alkylation by treatment with 50 mM iodoacetamide/0.1 M NH_4_HCO_3_ for half an hour in dark at 28 °C. After the treatment, gel pieces were washed with 25 mM NH_4_HCO_3_ in 50% acetonitrile. Thereafter the gel pieces were treated with trypsin digestion buffer (50 mM NH_4_HCO_3_, 5 mM CaCl_2_, 12.5 ng/μ l trypsin) as described earlier^1^.

### Identification of protein by MALDI-TOF MS

The peptide digest extracted from the gel piece (1 μ l) was premixed with same volume of the matrix and spotted on a matrix-assisted laser desorption ionization (MALDI) plate. The Peptide mass fingerprint (PMF) was analyzed using MALDI TOF- mass spectrometer in the reflector mode (Ultraflex II, Bruker Daltonics, Germany). Swiss-Prot database using MASCOT search engine (Matrix Science, London, United Kingdom) with a peptide mass tolerance of 100 ppm was used to search the data generated.

### Energy Dispersive X-ray Spectroscopy (EDAX) analysis and FEG-SEM of archaeal ionic composition

The accumulation of intracellular ions in response to stress was studied using EDAX analysis^32^. *Hal. marismortui* RR12 was cultured in SG broth containing 15% NaCl and incubated at 40 °C at 120 rev min^−1^. After appropriate incubation, the cells were harvested by centrifugation at 10,000 ×  *g* for 30 min and then exposed to stress by inoculating in SG medium containing 8% and 25% NaCl for 24 h at 40 °C. After 24 h, the cells were centrifuged, dried and fixed using 2% glutaraldehyde and used for observation of change in morphology by Field Emission Gun Scanning Electron Microscopy (FEG-SEM) and the intracellular ions were determined by Energy dispersive X-ray spectroscopy (EDAX) (Jeol, Japan).

### Detection of intracellular ion concentration using ICP-AES

As the intracellular sequestration of sodium ions was expected during NaCl stress, the intracellular concentration of sodium ions in cells exposed to varying concentrations of NaCl was performed as described by Jensen *et al.*^6^. Briefly, cells were cultured in SG medium containing 8% (1.36 M) NaCl, 15% (2.5 M) NaCl and 25% (4.2 M) NaCl. The cells were pelleted by centrifugation at 10,000 ×  *g* for 10 min and the media was removed. The cells were lysed using nitric acid treatment and the intracellular sodium ions were detected using Inductively Coupled Plasma Atomic Emission Spectroscopy (ICP-AES) (SPECTRO Analytical Instruments, GmbH, Germany).

### Extraction of protective carotenoid produced in response to salinity stress

The production of red pigment produced in response to salinity stress in *Hal. marismortui* RR12 was studied. The organism was exposed to varying concentrations of NaCl in SG medium as described earlier. The total pigment content present was estimated by measuring the absorbance of the culture broth at 490 nm using UV-Vis Spectrophotometer (Shimadzu, Japan)^33^.

## Results

Salterns are shallow ponds that comprise of hypersaline thalossohaline ecosystems that utilize natural evaporation process for production of common salt during summer season. Sea water is allowed to enter the shallow ponds and is retained for evaporation^12^. Previously, haloarchaea have been isolated from salterns of Mulund area, Mumbai^34^, salterns of Ribander, Goa^12^ and salterns of Kanjur Marg area from Mumbai by our group^16^. However, this is probably the first report of isolation of halophilic archaea from salterns lining Bhandup area of Mumbai, India. The elemental analysis of the brine and saltern soil was done by standard methods (Table 1) and compared to the elemental analysis of some of the representative brine samples globally^15^.

The trend in concentration of ions was found to be Cl^−^ > Na^+^ > Ca^+2^> Mg^+2^ > K^+^. The sodium ion content in Bhandup saltern soil sample was found to be 46.55 g/L which is similar to the ion concentration of Greater Rann of Kutch, India^15^. The soil lining the saltern of Bhandup also contained more calcium deposits and magnesium deposits as compared to other hypersaline environments. This indicates that the organisms thriving in the Bhandup saltern ecosystem survive in the presence of abundant ion rich environment.

Out of 34 haloarchaeal strains isolated from Mumbai salterns, a total of nine haloarchaea were selected on the basis of tolerance to salinity and morphological as well physiological characteristics for further studies. The organisms were identified on the basis of standard biochemical test as well as 16S rRNA gene sequencing (Table 2).

**Figure 1:**
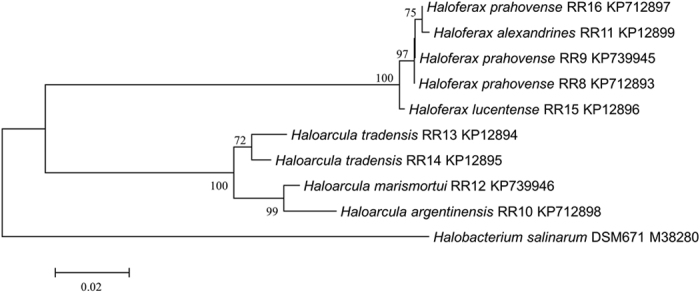
Construction of phylogenetic tree based on 16S rRNA gene sequencing by neighbor joining method. The evolutionary distances were computed using the Tamura 3-parameter method and are in the units of the number of base substitutions per site. The rate variation among sites was modelled with a gamma distribution (shape parameter =  1). The analysis involved 10 nucleotide sequences. Evolutionary analyses were conducted in MEGA 6.

The genera *Haloferax* and *Haloarcula* were predominant among the isolated haloarchaea. It was interesting that only these two genera dominated the saltern during the salt crystallization stage when the salinity was extremely high. Similar results were obtained by others who reported the dominance of *Haloferax*, *Haloarcula* and *Halorubrum* in the saltern during salt harvesting phase and the dominance of *Halococcus* during initial low salinity phase^12^. On the basis of 16S rRNA gene sequencing, biochemical and morphological characterization, the isolates were identified as *Hal. marismortui* RR12, *Hal. tradensis* RR14, *Hal. argentinensis* RR10, *Hfx. lucentense* RR15 and *Hfx. prahovense* RR9. To the best of our knowledge, these Haloarchaea have been reported for the first time from Bhandup salterns of Mumbai, India. The evolutionary relationship between the organism in the current study and other organisms is given in Fig. 1.

The haloarchaeon RR12 was identified as *Hal. marismortui* and it belongs to the family *Halobacteriaceae,* which consists of extremely halophilic organisms. This organism was isolated from evaporation ponds of sea water near Alicante, Spain, for the first time^35^. *Hal. marismortui* RR12 produced amylase, oxidase, catalase, casease, gelatinase and hydrolyses Tween 80. It produces intense red pigmentation and also demonstrates anaerobic growth in presence of arginine and DMSO. The membrane protein bacteriorhodopsin was also detected in *Hal. marismortui* RR12. The unique characteristic of this organism is that it has been found that this organism exhibits rapid growth than other members of *Halobacteriaceae* and demonstrates excellent genome stability and metabolic efficiency even at high salt concentrations. Because of the outstanding stability of this organism, it has proved as a good model to study haloarchaeal metabolism and physiology studies for several years^36^. The novel characteristic of *Hal. marismortui* RR12 is its ability to adapt to rapid changes in NaCl concentrations and survive despite the large fluctuations. This makes the organisms suitable to study the effect of salinity stress as well as for production of compounds pertaining to biotechnological as well as pharmaceutical significance. Hence, *Hal. marismortui* RR12 was chosen for further experiments related to salinity stress.

**Figure 2:**
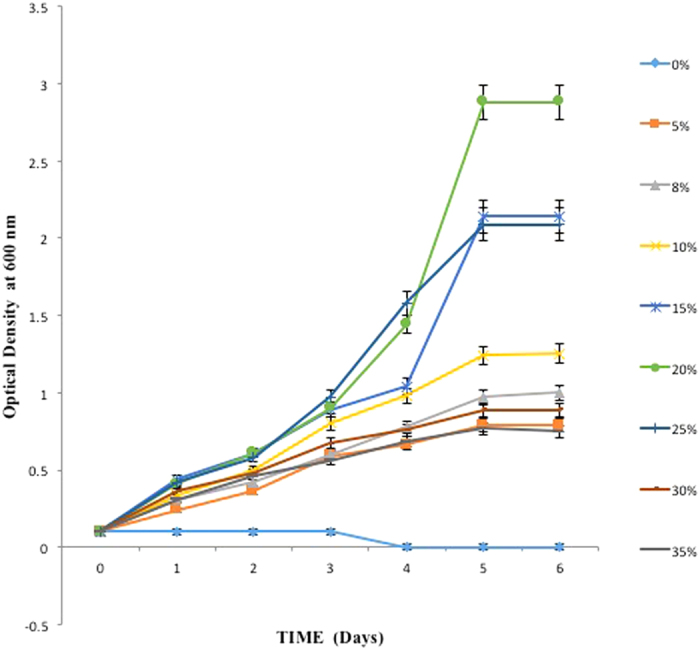
Effect of salinity concentration on growth of *Hal. marismortui* RR12. Bar represents standard error (SE).

The effect of different salinity concentrations in the range of 5%, 8% 10%, 15%, 20%, 25%, 30% & 35% on growth of *Hal. marismortui* RR12 was studied and the results are depicted in Fig. 2. Figure 2 indicates that the organism demonstrated extremely good growth at 15% and 25%. The haloarchaea is not capable of growth in medium without sodium chloride (0% NaCl). Growth of the organism was lesser at 5%, 8%, 10%, 30% and 35% NaCl with generation time as 72 h, 48 h, 50 h, 48 h and 70 h respectively. The isolate showed optimum growth in the range of 15 to 25% NaCl with a generation time of 24.5 h, 24 h and 24.3 h for 15, 20 and 25% NaCl respectively. This shows that though the growth is lesser at extreme low or high salinity, Hal*. marismortui* RR12 is capable of demonstrating growth over a wide range of sodium chloride concentrations and the study of the plausible adaptation strategies developed in this organisms may provide interesting insights in the physiology and survival mechanisms of Haloarchaea. To explore the adaptation response of the isolate at hypo salinity and hyper salinity, sodium chloride concentrations of 8% (low salinity) and 25% (high salinity) were selected for further.

The differential expression of proteins produced by *Hal. marismortui* RR12 was investigated using a non targeted proteomic approach. The bands that were predominantly present in samples with hypo or hyper salinity stress were characterized by MALDI-TOF MS (Table 3).

The proteins expressed in hypo salinity were, transcriptional regulatory protein rrnAC2519 and GTP cyclohydrolase III. The GTP cyclohydrolase are metal dependent enzymes involved in the biosynthesis of certain vitamins and cofactors^37^. The transcriptional regulatory protein rrnAC2519 is a Helix-turn-helix (HTC) type transcription regulatory protein containing a DNA binding motif. It is involved in the biological process of transcription in Haloarchaea with specifically in regulating gene expression via protein-DNA interactions^38^. In response to hyper salinity, ORC1-type DNA replication protein 1 OS and Putative Flagella related protein were detected. The ORC1-type protein is a DNA replication protein and a part of the DNA replication apparatus in Haloarchaea^39^. The relative abundance of accumulation of different cations and anions in contrasting salinities is shown in Table 4.

**Figure 3:**
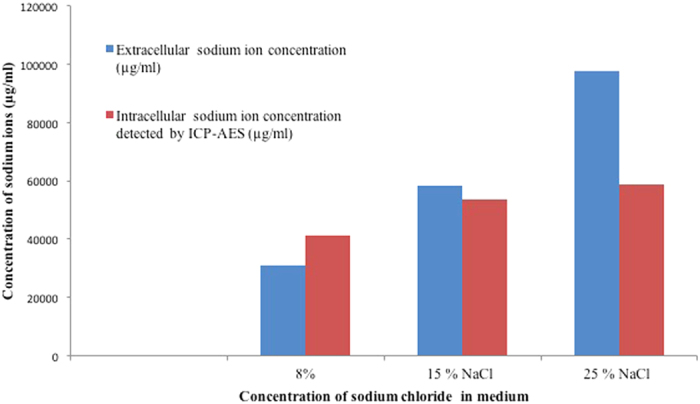
Detection of intracellular sodium ion accumulation by ICP-AES in *Hal. marismortui* RR12 cultured in SG broth containing varying concentration of NaCl.

It is evident from the EDAX analysis that the accumulation of intracellular ions in archaea varied with external salt concentration. It is apparent from the EDAX analysis, that potassium was the dominant ion sequestered in hyposalinity and showed highest accumulation at 8% NaCl conditions. At 25% NaCl concentration, chloride sequestration was higher and a trend of chloride > sodium > potassium > phosphate was obtained whereas at 8% NaCl the trend changed to potassium > Sodium > chloride. From the analysis it was observed that *Hal. marismortui* RR12 accumulates chloride ions in higher salt concentration and sequesters potassium ions at low salt concentrations that indicates the mechanism of “salt-in” strategy of osmoadaptation in response to contrasting salinities. It was intriguing that in contrasting NaCl stress, sodium was not the preferred ion for maintenance of stable concentration gradient. Hence, the role of sodium ion in osmoadaptation during NaCl stress was further investigated using the highly sensitive technique of ICP-AES. It was observed that in hyposalinity, intracellular sodium ion concentration was higher than extracellular sodium ion concentration. In hypersalinity, though the extracellular ion concentration is high, the cell maintains a low intracellular concentration of sodium ion. (Fig. 3).

The salt-in mechanism is a common haloarchaeal strategy of osmoadaptation. It is known in haloarchaea, that despite the relative abundance of sodium ions in the extracellular sink, these organisms tend to accumulate potassium instead of sodium via an energy dependent potassium uptake system^6^.

Salinity stress was found to notably affect cellular morphology of *Hal. marismortui.* Although, irregular pleomorphic forms are common morphological types of haloarchaeal cells. *Hal. marismortui* demonstrated changes in morphology in contrasting salinities. The cells of *Hal. marismortui* RR12 demonstrated significant elongation and appeared slender when exposed to low salinity (8% NaCl). When the cells were exposed to higher salinity, the cells swelled up and appeared thicker. The changes in morphology demonstrated that the flux in salinity not only affects the intracellular ion concentration and proteome of the bacteria but also causes variations in cellular morphology (see Supplementary Fig. S1).

*Hal. marismortui* RR12 produces typical red pigment in broth and agar cultures. It was observed that when the organism was exposed to increasing salinity stress, the production of red pigment increased as evidenced in increase in absorption at 490 nm (see Supplementary Fig. S2). The spectral scan of the extracted pigments showed characteristic absorption peaks of carotenoids as evidenced by maximum peaks between 400–490 nm. This indicated the possible presence of β -carotene, phycotene, lycopene or bacterioruberin. The red pigmentation in haloarchaea is known to be due to the membrane bound carotenoids and C50 bacterioruberin and other derivatives. The pigments of haloarchaea are known to play a protective role in maintaining cell homeostasis as well as in protection against oxidative damages and other stresses.

## Discussion

Haloarchaea are a group of organisms that have evolved dynamically in terms of their physiology for adjusting, adapting and thriving in the presence of multiple stresses. The survival of these organisms in salt sediments of the late Permian and early Triassic era (ca. 240–280 million years ago) has been studied. Dombrowski and Reiser and Tasch have previously described the isolation of viable organisms from ancient rock salts^40^. Similarly, *Haloarcula* sp. have been isolated from British salt mines of the Permian and Triassic age^41^. The survival of these organisms in these salt deposits is extremely interesting in terms of long term survival of microorganisms and studies related to origin of life. Likewise, the growth and endurance of these haloarchaea in hypersaline thalossohaline salterns is extremely remarkable as these environments are dynamic ecosystems and niches with constant flux in concentrations of sodium chloride.

The focus of the current study was to isolate haloarchaea tolerant to hyper salinity and expose it to hypo salinity and study the response of the isolate to the extreme differences in NaCl concentrations. Hence the previously unexplored salterns of Bhandup, Mumbai were chosen as sampling sites as its ionic analysis revealed that the sodium ion concentration in the brine was greater than the concentration of sodium ions in Dead Sea and comparable to sodium ion concentration in Great Salt Lake, USA^15^. The studies related to physiological survival of *Haloarcula* in contrasting salinities (hypo and hyper salinity) are still unprecedented and this study aimed at investigation of the response of NaCl stress on haloarchaea. Out of 34 isolates from Mumbai salterns, 9 were identified and the predominant genera obtained were *Haloferax* and *Haloarcula. Hal. marismortui* RR12 strain adapted and demonstrated growth in varying sodium chloride concentrations and hence was selected for further studies.

The two major physiological strategies for stress survival in Haloarchaea are accumulation of intracellular compatible solutes and “salt-in strategy”. Most haloarchaea accumulate compatible solutes for maintaining cell turgor pressure. Compatible solutes are organic low molecular weight compounds like betaines (glycine betaine), amino acid (proline, glutamine), polyols (sorbitol, mannitol), sugars (sucrose, trehalose) and ectoines (ectoine and hydroxyectoine)^42^. The compatible solutes have an osmoprotective effect and also serve as protectants against high temperature, freezing, desiccation and toxic radical stress. In nutrient starvation, these organic osmolytes also serve as carbon, nitrogen and energy sources^7^. In halophilic archaea, de-novo synthesized nitrogen compounds serve compatible solutes and they adapt to low water-potential habitats by stabilizing structural dense and less dense fraction of cellular cytosol by fitting in lattice of free water thereby facilitating the formation of *in situ* hydration cluster^43^. The organic solute strategy has been largely explored earlier and hence we focused on investigations related to the second stress survival strategy, viz. “salt-in strategy” in addition to exploring the proteins produced in response to stress.

The “salt- in strategy” is a classical strategy of halophilic archaea in which the organism raises the salt concentration by intracellular accumulation and sequestering of ions into the cytoplasm so as to thermodynamically adjust the cell to varying concentration gradient of the salt across the cell wall. Despite the relative abundance of sodium ions in the extracellular environment, the organism resorted to intracellular accumulation of K^+^ ion through an energy dependent potassium uptake system. Though the intracellular K^+^ ion concentration is higher than Na^+^, as the cells enter stationary phase, K^+^ is gradually replaced by Na^+ ^44. *Hal. marismortui* RR12 uses the salt-in strategy for survival in stress conditions^6^. When the organism was exposed to low salinity, it sequestered K^+^ ions (Table 4). The previously reported ATP -regulated K^+^/H^+^ symporter identified in *Haloarcula marismortui*^6^ may be involved in potassium ion transport of the isolate RR12.

When exposed to hyper salinity, the accumulation of Cl^−^ ions at higher salinities was observed. In both hypo and hyper salinity, sodium was not the preferred ion sequestered intracellularly in *Hal. marismortui* RR12. Thus in salinity stress, the haloarchaeon accumulates multiple ions as a part of its dynamic salt-in mechanism. The proton motive force plays a pivotal role in balancing the fluctuating ion gradients across membrane and it also supplies energy to the ATP driven ion pumps for the ion efflux as well as sequestration.

Majorly, organisms of the order Halobacteriales utilize proton electrochemical gradient to drive sequestration of potassium and expulsion of sodium ions. Maintenance of gradient is by aerobic respiratory electron transport, hydrolysis of ATP via membrane ATPases or by the photosensitive bacteriorhodopsin mediated proton pump^45^. The established proton pump is then used in juxtaposition with Na^+^/H^+^ antiporters to maintain sodium ion gradient across the membrane. Though salt in strategy with reference to potassium ion accumulated have been reported^6^,^46^, data related to sodium accumulation in *Hal. marismortui* is meager.

Similarly effect of increasing sodium ion concentrations on cellular morphology of *Haloarcula* sp. has been rarely explored. The Surface layer (S-layer) is involved in maintenance of cellular morphology of haloarchaea. The S-layer is a tightly packed hexagonal lattice of glycoproteins that requires cations for maintaining its stability^37^. It was observed that when *Hal. marismortui* RR12 was exposed to low salinity, the cells change its morphology. This is because the cell envelope layer of haloarchaea requires higher concentrations of cations for maintaining cell shape. The salt concentration thus alters the cellular morphology of the bacteria and though it does not cause lyses of cell at low concentrations, higher concentrations tend to stabilize the cell morphology.

Production of protective proteins by upregulation genes of responsible production of stress proteins is a common strategy in organisms. The common archaeal stress proteins are Hsp40 (*dnaJ*), GrpE (*grpE)* and Hsp70 (*dnaK)*^5^. Besides Haloarchaea also possess group II chaperonin class of molecular chaperones that are oligomeric proteins encoded by genes *cct1, cct2* and *cct3*^20^. Proteasomal components also play a fundamental role in major stress conditions in a ubiquitin free archaeal cytosol. *Hfx. volcanii* mutant strains (deletions in *psm*A and/or *pan*A) deficient in synthesis of proteasomal proteins were unable to survive in hypo saline environments of 1.6–1.8 M NaCl^21^. There are many previous reports on the involvement of these chaperonin, proteasomal proteins and stress proteins in haloarchaea exposed to different environmental stresses^1^,^5^,^19^,^20^,^21^. However besides these classical proteins, an intriguing group of small heat shock proteins (sHsp) of around 30 kDa or less are also involved in adaptation of stress in haloarchaea^5^. Though their role in eukaryotes and bacteria has been clearly identified, the study of these small molecular weight proteins in haloarchaea have been largely overshadowed to greater emphasis on studies related to the chaperonin systems in *Haloarchaea.* The proteins differentially expressed in response to hyper salinity and hypo salinity stress in *Hal. marismortui* RR12 were studied. In hypo salinity stress, two proteins were detected by MALDI-TOF MS in *Hal. marismortui*, viz. Transcriptional regulatory protein rrnAC2519 and GTP cyclohydrolase III of molecular mass 35. 6 kDa and 30.26 kDa respectively. The molecular weights of these proteins are in the range of the sHsp group and may find similarity in terms of functioning to the 17 unidentified proteins expressed in hypo salinity stress in *Hfx. volcanii*^5^. The environmental perturbations in haloarchaeal culture conditions in terms salinity stress need to be balanced genetically. Transcription factors (TF) are sovereign regulators of gene clusters and play a cardinal role in maintaining cellular homeostasis by binding to the promoters of genes responsible for production of stress protectant proteins. Transcriptional regulatory protein detected in *Hal. marismortui* is a Helix-turn helix type DNA binding protein cognate to the PF04937 domain family that plays a paramount function in regulation of gene expression via protein- DNA interactions^47^. Similarly, the second protein identified in response to hypo salinity was GTP cyclohydrolase III is involved in synthesis pathway of riboflavin^36^. The families of GTP cyclohydrolase are metal dependent enzymes involved in the biosynthesis of certain vitamins and cofactors that in turn may be involved in stress response.

In response to hyper salinity in *Hal. marismortui*, ORC1-type DNA replication protein 1 OS and Putative Flagella related protein were detected (Table 4). The flagella related protein is a putative protein which is a part of the archael flagellar apparatus made of flagellin protein coded by *flaC* gene. However, the exact role of *fla* proteins is not clearly understood though their role in flagellation is obvious. The ORC1-type protein is a DNA replication protein and a part of the DNA replication apparatus in Haloarchaea. The ORC protein contains N-terminal AAA +  domain and C-terminal winged-helix domain. Haloarchaea contain multiple origins of replications out of which many are dormant. These dormant origins of replications can be activated as a cellular and molecular response to caused by environmental stresses resulting in DNA replicative stress^48^. Besides, as a cellular adaptation mechanism, the cytosol of haloarchaea contains more acidic amino acids to balance the increased intracellular ion concentration. The sequence coverage data generated after MASCOT analysis of the proteins differentially expressed in salinity stress in *Hal. marismortui* indicated the presence of acidic amino acids in the peptides (Aspartic acid and Glutamic acid) that is also a stress adaptation mechanism. The increased charged amino acids on the proteins in turn stabilize the hydration shell of the molecules in presence of higher ionic strength containing cytosol^49^.

Haloarchaeal proteomes are highly complicated as they have to adapt to high concentrations of intracellular salt in which charged amino acids on the protein surface allow the retention of water molecules for catalytic activity^50^. This makes the haloarchaeal proteomes very acidic and depleted in lysine residues that makes the protein extraction from cell and sample preparation difficult. This affects the depth of sample coverage by isoelectric focusing, isotope-coded protein labeling (ICPL) of free amino groups and other proteomic approaches^51^,^52^. Besides, it is known that the proteome of *H. marismortui* is also highly acidic with an average isoelectric point of 5.0^38^. Thus when proteins of haloarchaea are separated using 2D-PAGE, they tend to aggregate and cluster in the narrow pI range of 4–5^53^. This makes the separation of protein spots on 2D gels difficult and this is a common problem associated with the study of archaeal proteomes^54^. Kirkland *et al.*^55^ identified 61 trans membrane domain containing proteins in *Hfx. volcanii*. However, none of these proteins could be detected in 2D gels. To overcome the drawbacks of 2D-PAGE, a combination of other modified approaches including strong cation exchange (SCX) chromatography coupled with reversed phase (RP) HPLC, tandem mass spectrometry (MS/MS) using nano-electrospray ionization hybrid quadrupole time-of-flight (TOF) (QSTAR XL Hybrid LC/MS/MS System) and quadrupole ion trap (Thermo LCQ Deca) was used for overall protein identification^55^. As a result of the evident drawbacks of the application of 2D gels for studying haloarchaeal proteomes, we used a simple modified approach in which we directly subjected the differentially expressed protein bands obtained in 1D SDS-PAGE to in-gel trypsin digestion followed by identification of the the peptide mass fingerprint generated using MALDI-TOF MS. As this study was based on general physiological mechanisms, this approach suited the current investigation, however for a deeper insight in the proteome of *Haloarcula,* other high-throughput methods or a combination of different methods like SCX with RP-HPLC followed by MS/MS or 2-D followed by MS/MS or LC-MS can be used in future.

The effect of salinity on pigmentation of the haloarchaeon was investigated. It was observed that when salinity increased, the pigment production also increased proportionally. Most haloarchaea produce pigments of varied colors (pink, red, purple, yellow) that are compounds of retinol or isoprenoid derivatives. The red color pigment is due to the production of carotenoids and bacterioruberin. These pigments are important as they play a pivotal role in protection of the haloarchaea in stress and also are involved in maintaining the fluidity of membrane besides being involved in photoresist system^56^. The production of excessive red pigment in response to increasing concentrations of salinity is indicative of the role of pigment as one of the physiological means of combating stress.

Overall, the cellular response of *Hal. marismortui* isolated from a hypersaline thalossohaline saltern of Bhandup, Mumbai to contrasting hypo and hyper salinities was studied. There are numerous known mechanisms of stress adaptations in haloarchaea. However, to the best of our knowledge, the current investigation is one of the first reports on understanding the concomitant cellular strategies like preferential sequestrations of ions validating ‘salt -in strategy’, involvement of sHsp and protective pigments as a response to contrasting salinity stress in *Hal. marismortui* RR12.

## Additional Information

**How to cite this article**: Thombre, R. S. *et al.* Biology and survival of extremely halophilic archaeon *Haloarcula marismortui* RR12 isolated from Mumbai salterns, India in response to salinity stress. *Sci. Rep.* **6**, 25642; doi: 10.1038/srep25642 (2016).

## Supplementary Material

Supplementary Information

## Figures and Tables

**Table 1:** Elemental analysis of representative brine samples across the world.

No.	Place of collection	Na^+^	K^+^	Cl^−^	Ca^+2^	Mg^+2^
1	Bhandup saltern, Mumbai, India (Brine)	94.05	nd	211.28	nd	8.26
2	Bhandup saltern, Mumbai, India (soil)	46.55	0.75	262.3	76.0	24.3
3	Great Rann of Kutch, India	46.0	2.0	157.0	Trace	16.1
4	Great Salt Lake (USA)	105.5	6.7	181.0	0.3	11.1
5	Dead Sea	40.1	7.6	225.0	nd	nd
6	Wadi Natrun (Egypt)	142.0	2.3	155.0	nd	nd

Values are expressed as g L^−1^. Data for Bhandup saltern soil and brine, Mumbai, India are from current study. (Nd, not determined).

**Table 2:** Identification of the haloarchaeal cultures using 16S rRNA gene sequencing.

Isolate No.	NCBI GenBank Accession no.	Nearest phylogenetic match	Identity (%)
RR8	KP712893	*Haloferax prahovense* TL6(T)	100
RR9	KP739945	*Haloferax prahovense* TL6(T)	99.38
RR10	KP712898	*Haloarcula argentinensis* JCM 9737(T)	98.42
RR11	KP712899	*Haloferax alexandrinus* TM(T)	98.64
RR12	KP739946	*Haloarcula marismortui* ATCC 43049(T)	99.14
RR13	KP712894	*Haloarcula tradensis* HST03(T)	97.00
RR14	KP712895	*Haloarcula tradensis* HST03(T)	97.67
RR15	KP712896	*Haloferax lucentense* JCM 9276(T)	99.18
RR16	KP712897	*Haloferax prahovense* TL6(T)	99.13

**Table 3:** Description of the differentially expressed protein in response to salinity stress.

S. No.	Description of peptide/protein	Organism	Gene	Inducer/Stressor	Reference
1	Transcriptional regulatory protein rrnAC2519^a^	*Haloarcula marismortui* RR12	*rrnAC2519*	Salinity	Present study
2	ORC1-type DNA replication protein 1 OS^b^	*Haloarcula marismortui* RR12	*cdc6A*	Salinity	Present study
3	GTP cyclohydrolase III^a^	*Haloarcula marismortui* RR12	*gch3*	Salinity	Present study
4	Putative Flagella related protein^b^	*Haloarcula marismortui* RR12	*flaC*	Salinity	Present study
5	Transcription regulator	*Haloferax volcanii*	*psp A*	Salinity	Bidle *et al.*^22^
6	Ribosomal protein S7	*Haloferax volcanii*	*rpsG*	Salinity	Bidle *et al.*^22^
7	Ribosomal protein S7	*Haloferax volcanii*	–	Salinity	Bidle *et al.*^22^
8	Chaperonin protein	*Haloferax volcanii*	*cct1, cct2, cct3*	Temperature	Macario *et al.*^5^
9	Superoxide dismutase	*Halobacterium halobium*	*sod*	Heat	Bergonia *et al.*^47^
10	Bacterioruberin	*Haloferax medditerranei*	–	Osmotic stress	D’souza *et al.*^48^

Proteins are identified based on Peptide mass finger print (PMF) data acquired on the MALDI TOF-MS Protein analyzer (Ultraflex II, Bruker Daltonics) in the reflector mode and compared to other protein expressed in stress reported in literature. ^a^Protein differentially expressed in response to low salinity stress (8%), ^b^Protein differentially expressed in response to high salinity stress (25%)].

**Table 4:** The relative abundance of total anions and cations in *Haloarcula marismortui* RR12 cultured in contrasting salinities.

S. No.	Ions	8% NaCl	25% NaCl
1	Na	3.45	1.88
2	Mg	1.89	1.03
3	K	7.20	1.88
4	Cl	2.68	3.01
5	P	1.35	1.88
