# Supplementary material for: Biology and survival of extremely halophilic archaeon Haloarcula marismortui RR12 isolated from Mumbai salterns, India in response to salinity stress
Source: Sci Rep. 2016 May 27;6:25642. doi: 10.1038/srep25642 (PMC4882750; doi:10.1038/srep25642)
Supplement: Supplementary Information [file srep25642-s1.doc]

Supplementary Information

**Biology and survival of extremely halophilic archaeon *Haloarcula marismortui* RR12 isolated from Mumbai salterns, India in response to salinity stress**

Rebecca S. Thombre^1^*, Vinaya D. Shinde^1^, Radhika S. Oke^1^, Sunil Kumar Dhar^2^ and Yogesh S. Shouche^2^

*Department of Biotechnology, Modern College of Arts, Science and Commerce, Shivajinagar, Pune-411005. Maharashtra, India.*

*^2^Microbial Culture Collection (MCC), National Centre for Cell Science, Ganeshkhind, Pune, 411007, India.*

**Supplementary Information Figure S1**


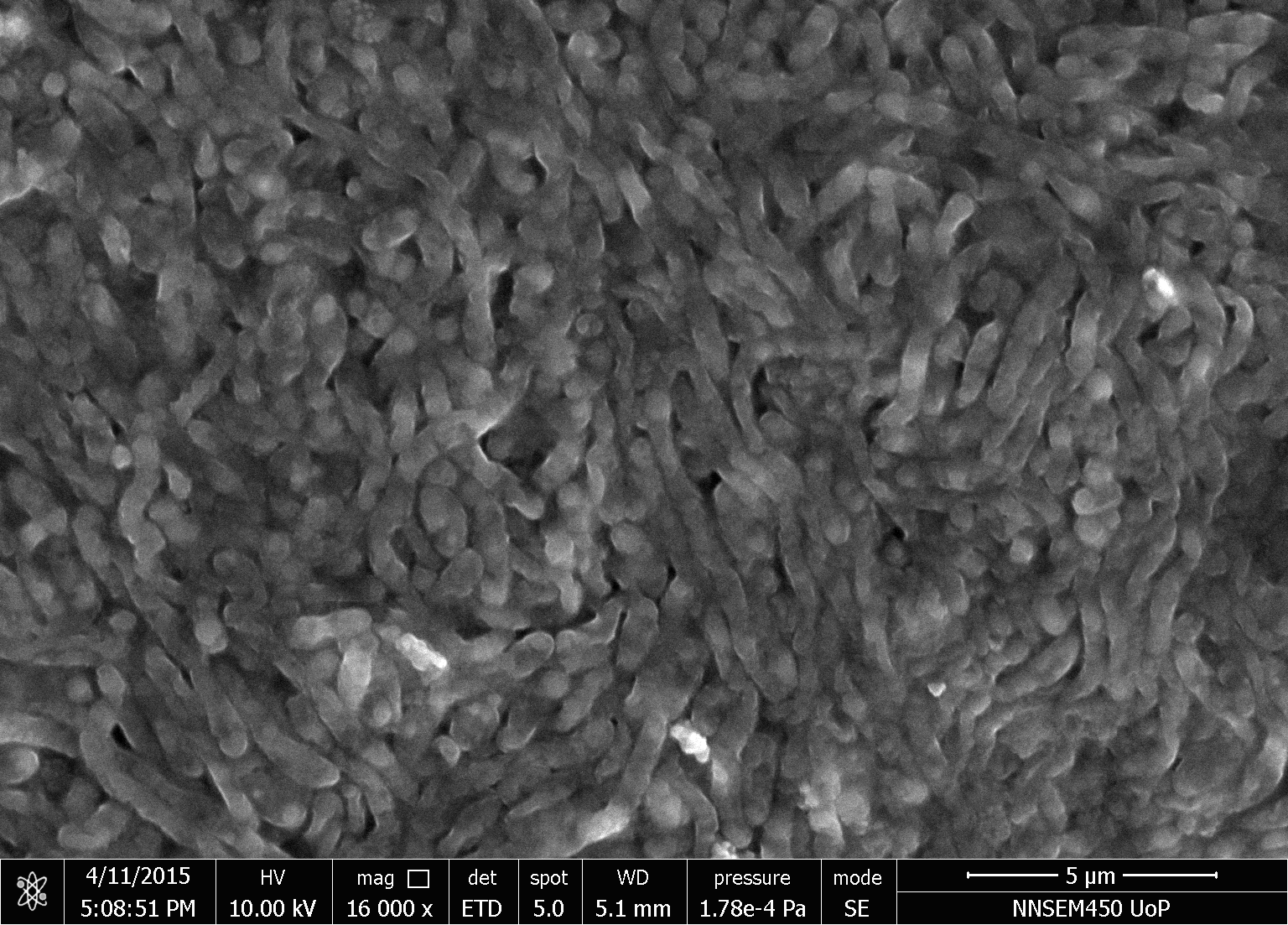

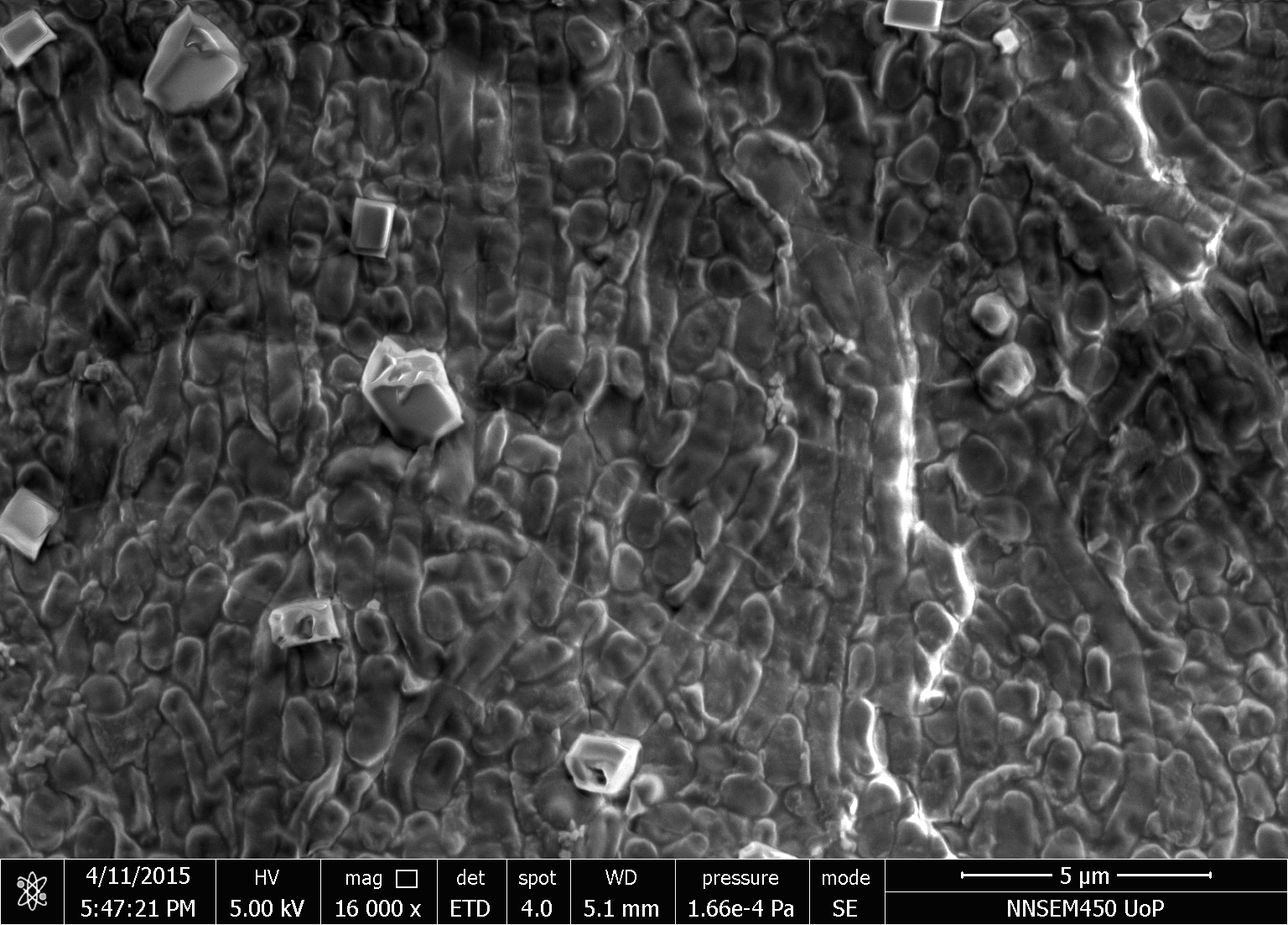


1. b.

**Fig. S1** Field Emission Gun-Scanning Electron Micrograph of *Haloarcula marismortui* RR12 exposed to, a: low salinity (8% NaCl) and b: high salinity (25% NaCl).

**Supplementary Information Figure S2**


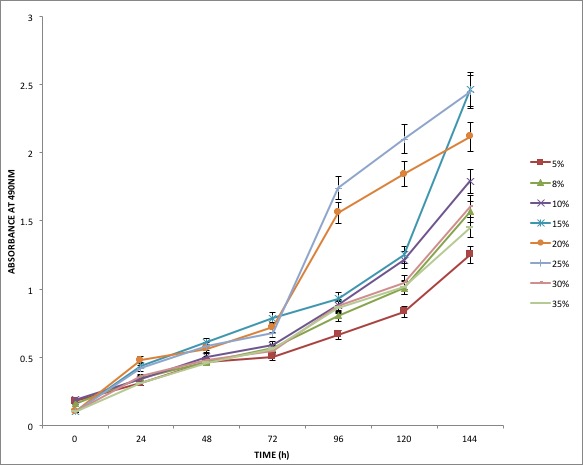


**Supplementary Fig. S2** Effect of salinity stress on pigment production by *Haloarcula marismortui* RR12
